# Cephalic Index in the First Three Years of Life: Study of Children with Normal Brain Development Based on Computed Tomography

**DOI:** 10.1155/2014/502836

**Published:** 2014-02-04

**Authors:** Wirginia Likus, Grzegorz Bajor, Katrzyna Gruszczyńska, Jan Baron, Jarosław Markowski, Magdalena Machnikowska-Sokołowska, Daniela Milka, Tomasz Lepich

**Affiliations:** ^1^Department of Human Anatomy, Medical University of Silesia, 18 Medyków Street, Bulding C-1, 40-752 Katowice, Poland; ^2^Department of Radiology and Nuclear Medicine, Medical University of Silesia, 12 Medyków Street, 40-752 Katowice, Poland; ^3^E.N.T. Department, Medical University of Silesia, 20-24 Francuska Street, 40-027 Katowice, Poland

## Abstract

Cephalic index is a highly useful method for planning surgical procedures, as well as assessing their effectiveness in correcting cranial deformations in children. There are relatively very few studies measuring cephalic index in healthy Caucasian young children. The aim of our study was to develop a classification of current cephalic index for healthy Caucasian children up to 3 years of age with normal brain development, using axial slice computer tomography performed with very thin slices (0.5 mm) resulting in more accurate measurements. 180 healthy infants (83 females and 97 males) were divided into 5 age categories: 0–3, 4–6, 7–12, 13–24, and 25–36 months. The average value of cephalic index in children up to 3 years of age amounted to 81.45 ± 7.06. The index value in case of children under 3 months was 80.19, 4 to 6 months was 81.45, 7 to 12 months was 83.15, in children under 2 years was 81.05, and in children under 3 years was 79.76. Mesocephaly is the dominating skull shape in children. In this study, we formulated a classification of current cephalic indices of children with normal brain development. Our date appears to be of utmost importance in anthropology, anatomy forensic medicine, and genetics.

## 1. Introduction

Development of a child's head depends upon the development of the brain [1, 2]. The brain reaches 90% of its size until the first year of age, while its complete development ends when the child turns 7 [2, 3]. Anomalies related to the shape of skull may develop in prenatal period or in the postnatal period, chiefly up to 2-3 years of age [3]. Among the deformations that develop in prenatal period or within the first months after birth, there are craniosynostoses [4, 5]. Cephalic index (CI) is an objective and highly useful parameter for determining the skull shape [6]. It is of use for surgeons and neurosurgeons, for assessing the pre- and postoperative correction of skull deformations [2, 5–7]. It is easy to determine and is highly repetitive. The index is indispensable for planning a surgical procedure and assessment of its effectiveness [8]. The notion of cephalic index (CI) was introduced by van Lindert et al. as percentage of width to length in any skull [6]. The width is defined as the distance between the most projecting points at the sides of the head, above and behind the ears; the length is the distance from the glabella and the most projecting point at the back of the head [1, 6, 7, 9–12]. One of the methods used for assessing the skull shape and determination of CI is the measurements of skull length and width, performed on computer tomography scans.

The research performed so far, comprising measurements of skull length and width in children, was carried out relatively long ago [8, 13–15] or concerned a different race than Caucasian [16–18]. Also, the specialist literature lacks new reports concerning the CI value in children under 3 years of age for white children with regular brain development. In connection with the above, using the highly precise tool, which is computer tomography, especially with the application of 0.5 mm width of scan slices (layers), the authors attempted to develop a classification of the current cephalic index for children under 3 years of age, with normal brain development.

## 2. Materials and Methods

### 2.1. Patients

The retrospective study was conducted using computer tomography scans, on 180 Polish children of Caucasian race (83 females and 97 males) with normal brain development, age range from 0 (immediately after birth) to 3 years of age. All the patients were diagnosed between February 2009 and January 2012 in the Department of Imaging Diagnostics (Radiology) and Nuclear Medicine, Medical University of Silesia, Katowice, Poland (Table 1).

Qualified for morphometric analyses were those children, who were referred for head CT due to suspicion of head injury. Only those tomograms have been included in the assessment of skull dimensions, for which no transgressions have been found as regards the normal condition of skull osseous structures. Excluded from the study were prematurely born children, children with changes within the area of skull bones, with diseases of genetic origin (e.g., Down's syndrome), mental deficiency, congenital anomalies and/or congenital syndromes (e.g., CHARGE association, VATER association, Apert's syndrome, Crouzon's syndrome, and Pfeiffer's syndrome), craniosynostosis, and hydrocephalus.

The study group was divided into 5 age categories: 0–3 months; 4–6 months; 7–12 months; under 2 years of age (13–24 months); under 3 years of age (25–36 months).

Approval for this retrospective study was obtained from Bioethical Commission of the Medical University of Silesia.

### 2.2. CT Protocol and Image Analysis

**Figure 1:**
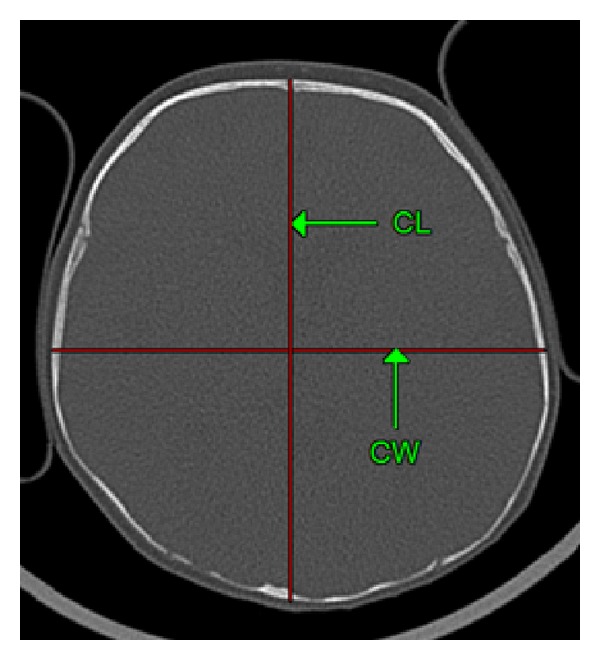
Measurements of the cranium (axial CT scan; child age: 5 months): CL: cephalic length, CW: cephalic width. Cephalic index = CW/CL × 100.

The computer tomography examinations were performed using spiral technique in transverse plane, in 0.5 mm slices (layers) using a 64-row scanner TOSHIBA Aquilion (Toshiba, Tokyo, Japan) applying the standard diagnostic protocol for head examination. The obtained axial images from CT were transferred to a workstation for analysis. The measurement plane was parallel to the so-called Frankfurt plane (auriculo-orbital plane). Each scan was measured based on reliable bony landmarks, selected to assess the cranial vault, as described by Waitzman et al. [13]. All measurements were performed directly on CT films, the accuracy of each measurement was up to 1/100 mm, and they were standardised to a 5 cm reference scale on each film. The cephalic index was calculated according to the following equation: (cephalic width/cephalic length) × 100 (Figure 1).

Cephalic length was the distance between the most anterior and posterior point of the outer table of the skull. The distance between the outer skull tables at the widest points of the skull was the cephalic width [13]. The measurements were performed in two independent series (repetitions). Results have been averaged.

### 2.3. Statistical Analysis

The consistence of the empirical distribution of variables examined with normal distribution has been assessed using the Shapiro-Wilk test. The homogeneity of variance has been assessed by means of Levene test. For the assessment of differences between the age groups studied, in terms of arithmetic means, the single-factor variance analysis (ANOVA) has been applied. In order to assess the significance of differences between sexes in a given age group, the Cochran Cox has been applied. The statistical difference between groups has been assessed at the level of *P* ≤ 0.05.

## 3. Results

The average value of cephalic index (CI) in children under 3 years of age in the study group amounted to 81.45 ± 7.06. Statistical analysis did not reveal statistically significant differences between specific age groups. In the group of girls under 3 years of age, the average CI amounted to 80.54 ± 7.20, while in the group of boys of the same age, 82.22 ± 6.87. No statistically significant differences were noted between sexes, as regards the value of CI (*P* = 0.027) (Table 2).

Minimum and maximum CI values are provided in Table 2. The minimum CI value for children under 3 years of age, with normal brain development, whatever the sex, amounted to 62.09, and the maximum amounted to 103.53 (Table 3).

In order to analyse the skull shape on the basis of cephalic index, for the purpose of this study, the classification according to Cohen and Maclean has been assumed [8]. Four categories of skull shape have been established: CI up to 75.9, dolichocephaly; CI: 76.0–80.9, mesocephaly; CI: 81.0–85.4, brachycephaly; CI: 85.5 or above hyperbrachycephaly.

**Figure 2:**
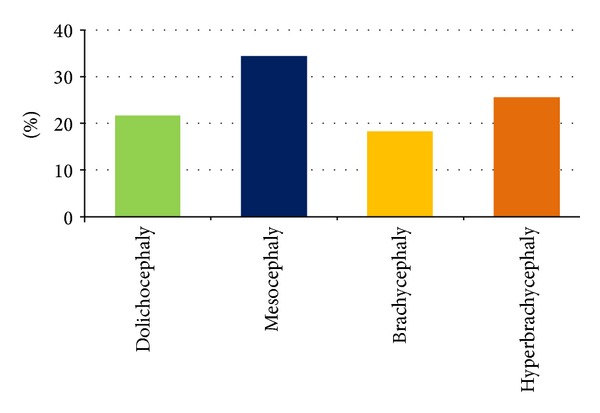
The shape of skull in children under 3 years of age according to Cohen's classification.

The analysis of skull shape in the study group of children under 3 years of age, in both sexes, indicated that the dominating type is mesocephalic shape (34%). Hyperbrachycephaly occurred in 26%, whereas dolichocephaly in 22% of the children. In the group examined, brachycephaly (18%) was the least frequently observed category (Figure 2).

**Figure 3:**
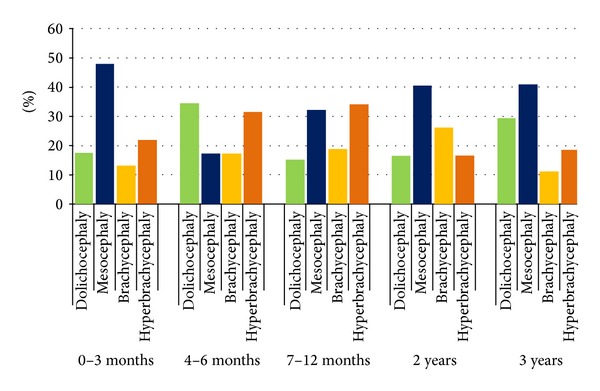
The shape of skull in children, according to Cohen's classification in different age.

The analysis of skull shape in specific age groups, for both sexes, indicated that, with the exception of infants between 4 and 6 months of age, the dominating skull shape was also mesocephaly (Figure 3).

More frequent occurrence of mesocephaly in females in comparison with males has been demonstrated, in the infants belonging to age groups: 4–6 months of age and 7–12 months of age (*P* = 0.029 and *P* = 0.045, resp.). Brachycephaly occurs more often in males than in females in case of infants from the age group of 7–12 months (*P* = 0.041). In the remaining age groups, no statistically significant differences have been noted, as to the frequency of occurrence of a given skull shape category, as classified by Cohen, and the sex of the child.

## 4. Discussion

Craniosynostosis or premature atresia of cranial sutures is a developmental disorder classified among the so-called bony face deformations [5, 8]. They may occur in isolated form or as part of syndromes [8]. Reduction of cranial cavity capacity may lead to compression of the normally developing child's brain. The plan of effective treatment in craniosynostoses comprises surgical treatment of skull deformation. For that purpose, the knowledge of skull dimensions of children with normal brain development is indispensable. One of the useful indicators for assessment of child's head shape is the determination of cephalic index, defined as percentage of width to length in any skull ((cephalic width/cephalic length) × 100%) [6]. The cephalic index may be determined applying anthropometric methods, dry skull measurements, and radiological methods (measurements on computer tomography scans). When analysing the child's head shape, it is important to tell the abnormal shape caused by positioning from that caused by pathologic processes, such as cranial sutures atresia. Flattening and asymmetry may have numerous reasons, the skull shape varies between individuals, being a combination of genetic and environmental factors [19, 20]. The reasons may range from uterine walls compressing fetus head to external reasons that occur after the birth, to which the newborn and infant are particularly exposed. Numerous authors point out that cranial deformations may result from the fact that infants, especially newborns, are invariably arranged in the same position when they are about to sleep [21, 22]. Babies call for intensive medical care; premature infants are particularly prone to such changes in skull shape [22]. According to Hummel and Fortado [20], CI values in newborns in dorsal position are between 86 and 88, while in case of lateral recumbent position, the value is between 76 and 81. CI values exceeding 81 mean shortening of the anteroposterior diameter, what results in brachycephaly. It should be remembered that infant's skull is highly plastic, and change of recumbent position may change and also improve the skull shape [22]. Graham et al. and Cartwright indicate that normal CI range for infants is 76–81, whereas for infants sleeping in dorsal position CI values much exceed 81 [21, 22]. Collett et al. assessed the shape of cranial basis and its asymmetry in infants from the day of their birth to one year of age. The studies indicated that the most frequent forms of deformation are plagiocephaly and brachycephaly, as well as their combinations [7]. In connection with the above, when analysing skull shape, particularly of young children, one should take into account the skull shape changes that are not due to pathologies.

Measurements skull length and width, for the purpose of this study, were performed using head CT scans, in very thin slices (layers), 0.5 mm thick, which makes the results we obtained highly accurate. The available literature reports measurements performed on CT scans with substantial slice thickness of 7 mm, with the use of older generation of tomographs, which could have influenced the results obtained [13, 15, 23]. An advantage of multirow computer tomography is also the short time of examination, which reduces the number of movement-related artefacts, in particular in case of extremely young patients [15]. Waitzman et al. [23] proved the advantage of morphometric examinations with the use of computer tomography imaging over direct measurements of the skull. With minimum differences (measuring error below 5%) in direct measurements of osseous structures of the skull and indirect measurements performed on CT scans of the same skulls, they demonstrated that measurements performed on CT scans are highly reliable. According to Posnick et al., the determination of cephalic index is a quantitatively useful method for comparing skull shape in patients before and after sagittal stenosis surgery [14]. Some studies concerning the determination of cephalic index and assessment of skull shape have been performed on children from other races than Caucasian [14, 16–18]. Due to the race differences, in skull shape, the results obtained by those authors cannot be related to the CI values obtained in our study.

Our results show that mean value of the index for Polish children with normal development of brain amounts to 81.45 ± 7.06. No differences have been observed between sexes as regards CI values, with the exception of infants in the age group of 7–12 months, where values are higher for males (Table 2). Farkas et al. measured the head width and length in Caucasian children directly from the head of the subject [9]. Their results indicate that CI values are as follows: in females and males under 1 year of age: 75.14 and 75.30, respectively; under 2 years of age: 76.24 and 75.80, respectively; and under 3 years of age: 75.32 and 75.30. In comparison with their results, obtained 21 years earlier, our study indicated far higher CI values, especially in children from the age range of 10-11 months, as well as under 1 and under 2 years of age (Table 2). Our results are convergent with the results of measurement performed on roentgenograms by Haas on children between 2 and 3 years of age [24]. We also obtained higher CI values, when compared with the results obtained by Weitzman et al. for Caucasians [13]. It should be underlined that Waitzman et al. measured the skull shape on scans from computer tomography made as dry skulls [13]. The results obtained in our study indicate that the value of cephalic index is the highest for children in the age range of 10–12 months (81.2) and gets reduced by the age of 3. With the cephalic index in use, the skull shape may be defined as dolichocephaly, mesocephaly, brachycephaly, and hyperbrachycephaly. When the skull shape is determined, we need to take into account the fact that various authors apply various CI ranges when defining a specific shape. Standring defines dolichocephaly for the CI value up to 74.9, mesocephaly: 75.0–79.9, brachycephaly: 80.0 to 84.9, and hyperbrachycephaly for CI exceeding 85 [1]. In accordance with Cohen's classification, the respective categories are as follows: dolichocephaly up to 75.9, mesocephaly: 76.8–80.9, and brachycephaly: 81.0–85.4, with hyperbrachycephaly classified at CI exceeding 85.5 [8]. Koizumi et al. introduced a classification according to which CI under 76 signifies dolichocephaly, and CI of 76–80.9 signifies mesocephaly, while CI exceeding 81.0 signifies brachycephaly [16]. Our research demonstrated, applying the skull shape classification according to Cohen, that the most frequently occurring type is mesocephaly (34%); however, if a different classification is used, namely, when groups with CI exceeding 81.0 are clustered together, brachycephaly (44%) would be the most frequently encountered skull shape. Collett at al. indicate that the shape of skull in children under 12 months of age is more brachycephalic [7]. This has also been confirmed recently by Hummel and Fortado for children from the USA [20]. Our studies indicate that mesocephaly is the dominating shape of skull in children below 3 years of age, which is also confirmed by the results obtained by Waitzman et al. [13].

## 5. Conclusions

Cephalic index is a useful and indispensable tool used for assessing skull shape in children, especially for the purpose of pre- and postoperative correction of skull deformations. The authors developed a classification of current cephalic index for Caucasian children under 3 years of age, with normal brain development.

## Figures and Tables

**Table 1:** Sex distribution of study population.

Age categories	Female (*n*)	Male (*n*)	Total (*n*)
0–3 months	13	10	23
4–6 months	20	15	35
7–12 months	27	26	53
<2 years	14	28	42
<3 years	9	18	27

Total	83	97	180

**Table 2:** Cephalic index in children with normal brain development (mean ± SD).

Age categories	Cephalic index total	Cephalic index female	Cephalic index male	*P* F versus M
	Mean ± SD	Mean ± SD	Mean ± SD	
0–3 months	80.19 ± 7.49	81.04 ± 7.48	79.09 ± 7.78	*P* = 0.549 (NS)
4–6 months	81.45 ± 7.98	79.54 ± 8.40	83.99 ± 6.84	*P* = 0.103 (NS)
7–12 months	83.15 ± 7.98	80.80 ± 7.37	85.59 ± 7.63	*P* = 0.027
<2 years	81.05 ± 5.24	81.99 ± 5.53	80.53 ± 5.12	*P* = 0.42 (NS)
<3 years	79.76 ± 5.56	78.98 ± 5.23	80.15 ± 5.82	*P* = 0.61 (NS)

	81.45 ± 7.06	80.54 ± 7.20	82.22 ± 6.87	

SD: standard deviation; NS: not significant.

**Table 3:** The range of cephalic index in children with normal brain development.

Age categories	Cephalic index total		Cephalic index female		Cephalic index male	
	Min	Max	Min	Max	Min	Max
0–3 months	62.08	96.82	72.19	96.82	62.09	87.99
4–6 months	69.57	98.40	69.58	98.41	70.29	93.18
7–12 months	66.50	103.55	66.50	96.06	69.91	103.53
<2 years	71.88	97.08	73.56	90.48	71.89	97.08
<3 years	72.49	90.87	72.49	90.88	73.97	90.88

	62.09	103.53	66.50	98.41	73.97	103.53

Min: minimum; Max: maximum.
